# Cross-Sectional Analysis of Late HAART Initiation in Latin America and the Caribbean: Late Testers and Late Presenters

**DOI:** 10.1371/journal.pone.0020272

**Published:** 2011-05-26

**Authors:** Brenda Crabtree-Ramírez, Yanink Caro-Vega, Bryan E. Shepherd, Firas Wehbe, Carina Cesar, Claudia Cortés, Denis Padgett, Serena Koenig, Eduardo Gotuzzo, Pedro Cahn, Catherine McGowan, Daniel Masys, Juan Sierra-Madero, on behalf of the CCASAnet Team

**Affiliations:** 1 Instituto Nacional de Ciencias Médicas y Nutrición, Salvador Zubiran, Mexico City, Mexico; 2 Biostatistics, Vanderbilt University, Nashville, Tennessee, United States of America; 3 Biomedical Informatics, Vanderbilt University, Nashville, Tennessee, United States of America; 4 Investigaciones Clínicas, Fundación Huésped, Buenos Aires, Argentina; 5 Fundación Arriarán, Universidad de Chile, Santiago, Chile; 6 Instituto Hondureño de Seguro Social y Hospital Escuela, Tegucigalpa, Honduras; 7 GHESKIO/Weill Medical College of Cornell University, Port au Prince, Haiti; 8 Brigham and Women's Hospital, Harvard Medical School, Boston, Massachusetts, United States of America; 9 Instituto de Medicina Tropical Alexander von Humboldt, Lima, Peru; 10 Infectious Diseases, Vanderbilt University, Nashville, Tennessee, United States of America; Instituto de Pesquisa Clínica Evandro Chagas/Fundação Oswaldo Cruz, Brazil

## Abstract

**Background:**

Starting HAART in a very advanced stage of disease is assumed to be the most prevalent form of initiation in HIV-infected subjects in developing countries. Data from Latin America and the Caribbean is still lacking. Our main objective was to determine the frequency, risk factors and trends in time for being late HAART initiator (LHI) in this region.

**Methodology:**

Cross-sectional analysis from 9817 HIV-infected treatment-naïve patients initiating HAART at 6 sites (Argentina, Chile, Haiti, Honduras, Peru and Mexico) from October 1999 to July 2010. LHI had CD4^+^ count ≤200cells/mm^3^ prior to HAART. Late testers (LT) were those LHI who initiated HAART within 6 months of HIV diagnosis. Late presenters (LP) initiated after 6 months of diagnosis. Prevalence, risk factors and trends over time were analyzed.

**Principal Findings:**

Among subjects starting HAART (n = 9817) who had baseline CD4^+^ available (n = 8515), 76% were LHI: Argentina (56%[95%CI:52–59]), Chile (80%[95%CI:77–82]), Haiti (76%[95%CI:74–77]), Honduras (91%[95%CI:87–94]), Mexico (79%[95%CI:75–83]), Peru (86%[95%CI:84–88]). The proportion of LHI statistically changed over time (except in Honduras) (p≤0.02; Honduras p = 0.7), with a tendency towards lower rates in recent years. Males had increased risk of LHI in Chile, Haiti, Peru, and in the combined site analyses (CSA). Older patients were more likely LHI in Argentina and Peru (OR 1.21 per +10-year of age, 95%CI:1.02–1.45; OR 1.20, 95%CI:1.02–1.43; respectively), but not in CSA (OR 1.07, 95%CI:0.94–1.21). Higher education was associated with decreased risk for LHI in Chile (OR 0.92 per +1-year of education, 95%CI:0.87–0.98) (similar trends in Mexico, Peru, and CSA). LHI with date of HIV-diagnosis available, 55% were LT and 45% LP.

**Conclusion:**

LHI was highly prevalent in CCASAnet sites, mostly due to LT; the main risk factors associated were being male and older age. Earlier HIV-diagnosis and earlier treatment initiation are needed to maximize benefits from HAART in the region.

## Introduction

Since the introduction of highly active antiretroviral therapy (HAART) for the treatment of HIV infection, the benefits in survival have been clearly established [1]. The stage of HIV infection at the time of HAART initiation plays an important role in patients' prognosis after treatment. Patients with advanced disease when starting HAART are less likely to achieve virological suppression, more likely to modify their therapy due to adverse events, have a higher mortality rate and represent a financial strain in public health services, as compared with those who initiate earlier [2]–[11]. Therefore, in recent years there has been a push towards earlier initiation of HAART to improve survival and decrease AIDS complications [12]–[15]. Furthermore, earlier HAART initiation may result in a lower risk of HIV transmission, as suppressing HIV-1 RNA levels has been shown to diminish transmission rates [16], [17]. As a result, early diagnosis and treatment of HIV constitutes a major public health issue.

Despite efforts to improve the care of people living with HIV, a considerable proportion of subjects do not obtain medical attention until they are in very advanced stages of the disease [8], [18]–[19]. Several studies conducted in high income countries in the past decade found that 20% to 40% of patients diagnosed with HIV were in advanced disease stages (in most studies defined as presenting with CD4 cell counts less than 200 cells/mm^3^ and/or a history of an AIDS-defining illness). Consequently, these patients were late HAART initiators [20]. Late HAART initiation can be attributed to late HIV diagnosis due to late testing (LT), or to late presentation (LP) to care after diagnosis has been established. Although late testing and late presentation may have similar consequences for the prognosis of individual patients when starting HAART [4]–[6], differences between LT and LP may reflect failures at different steps of health care access [21]. In Latin America and the Caribbean, studies show high frequencies of late stage of disease at HAART initiation [9]–[10], [22]–[23]. However, risk factors associated with LHI have not been well described. The aims of this study were to evaluate the proportion of patients who were LHI between the years of 2000 and 2010, to assess trends across the study period, to look for risk factors associated with LHI, and to determine if LHI was due to late diagnosis or late presentation using data from the Caribbean, Central and South American network for HIV Research (CCASAnet) [24].

## Methods

The CCASAnet cohort (http://ccasanet.vanderbilt.edu/) has been described elsewhere [9]–[10], [24]. The collaboration was established in 2006 as Region 2 of the International Epidemiologic Databases to Evaluate AIDS (IeDEA; www.iedea-hiv.org) with the purpose of collecting HIV data from Central and South America and the Caribbean to be able to describe the unique characteristics of the HIV epidemic in this region. Six CCASAnet sites contributed data to this study: Fundación Huésped in Buenos Aires, Argentina (FH-Argentina); Fundación Arriarán in Santiago, Chile (FA-Chile); Le Groupe Haïtien d'Etude du Sarcome de Kaposi et des Infections Opportunistes in Port-au-Prince, Haiti (GHESKIO-Haiti); Instituto Hondureño de Seguridad Social and Hospital de Especialidades in Tegucigalpa, Honduras (IHSS/HE-Honduras); El Instituto Nacional de Ciencias Médicas y Nutrición Salvador Zubirán in Mexico City, Mexico (INNSZ-Mexico); and El Instituto de Medicina Tropical Alexander von Humboldt in Lima, Perú (IMTAvH-Peru). Institutional review board approval was obtained locally for each participating site and for the CCASAnet data coordinating center (DCC) at Vanderbilt University, Nashville, TN, USA.

### Study design and population

The source population for this cross-sectional analysis was HIV-infected adults receiving medical care at the 6 participating CCASAnet sites. Treatment-naïve patients who started HAART between October 1999 and July 2010 were included. HAART was defined in all sites as all regimens with at least 3 antiretroviral drugs (mainly composed with 2 nucleoside/nucleotide analogs of reverse transcriptase inhibitors plus a no-nucleoside/nucleotide analogs of reverse transcriptase inhibitors or a protease inhibitor). All participant sites are part of local programs for universal free access to HAART. Late HAART initiators (LHI) were defined as those subjects with a non-missing baseline CD4 cell count (6 months before or 1 week after starting HAART) who either had baseline CD4≤200 cell/mm^3^ or an AIDS-defining illness (ADI) prior to or at HAART initiation. LHI were further classified as either late testers (LT) or late presenters (LP), corresponding to those who started HAART within 6 months of HIV diagnosis (LT) or more than 6 months after HIV diagnosis (LP). The remaining patients were considered non-late HAART initiators (NLHI). All study definitions were chosen a priori.

### Data Collection

Each participant site contributed data from their database using a standard data transfer protocol. Demographic, clinical, and laboratory data for all sites were prospectively collected and periodically recorded. The databases are updated routinely and sent to the DCC. Data were checked for errors and inconsistencies.

We collected data on sex, age at HAART initiation, probable route of infection (self-reported as sexual – either homosexual or heterosexual intercourse – or non-sexual), marital status (only available in GHESKIO-Haiti, INNSZ-Mexico and IMTAvH-Peru), employment status (employed/unemployed), and years of education at first clinic visit. In sites with available data (FH-Argentina and INNSZ-Mexico), sexual probable route of infection was further classified as homosexual or heterosexual. Baseline CD4 cell count was defined as the measurement closest to HAART initiation but not more than 6 months prior to, or 7 days after, the date of HAART start. Values not available within this time frame were considered missing.

### Statistical Analysis

Trends in the proportion of LHI across the study period were assessed non-parametrically using Friedman's ‘super smoother’ [25]. The association between LHI and risk factors was evaluated using univariate and multivariate logistic regression models. The primary multivariate analysis included age, sex, years of education, and date of HAART initiation – variables which were measured in all sites. Age and years of education were included as continuous variables. Secondary, site-specific multivariate models also included probable route of infection, marital status, and employment status, when available. Date of HAART initiation was included in the models using restricted cubic splines to account for non-linear time-trends [26]. Combined-site estimates were computed using random effects methods [27], [28]. Risk factors for LT and LP were assessed using multinomial logistic regression. In primary analyses, patients missing baseline CD4 cell count (LHI analyses) and baseline CD4 cell count or date of HIV diagnosis (LT/LP analyses) were excluded. Analyses were repeated incorporating data from all patients using multiple imputation techniques [29]. Statistical analyses were performed using STATA version 10 and R version 2.11.1.

## Results

A total of 9817 subjects started HAART during the study period. Patient characteristics stratified by site and combined are shown in Table 1 and Table S1 (Supporting Information section). The median age at HAART initiation was 36 years (interquartile range 30–43); 40% of patients were female, ranging from 55% in GHESKIO-Haiti to 11% in FA-Chile. The probable route of HIV infection was sexual for the majority of patients; only FH-Argentina had a substantial portion of patients whose probable route of infection was non-sexual. The mean years of education at first clinic visit was 8.6 (Standard Deviation 4.9) ranging from 6.1 years in GHESKIO-Haiti to 13.1 in FA-Chile. Twenty percent of patients were married, although marital status was only available in GHESKIO-Haiti, IHSS/HE-Honduras, INNSZ-Mexico and IMTAvH-Peru. Employment status at entrance to care was available in all sites except GHESKIO-Haiti; 73% of the cohort was employed. The median CD4 cell count at HAART initiation was 129 cells/mm^3^ (IQR 49–219); baseline CD4 cell count was missing for 1302 subjects (13%). The median time between HIV diagnosis and HAART initiation was 153 days (5.1 months; IQR 38–712 days); date of diagnosis was missing for 1836 (18.7%) subjects. The time period of the study differed between sites, although all sites contributed data from 2003–2008.

**Table 1:** Patient characteristics at time of HAART initiation by site.

	FH-Argentina	FA-Chile	GHESKIO-Haiti	IHSS/HE-Honduras	INNSZ-México	IMTAvH-Perú	Combined
	N = 1642	N = 931	N = 4458	N = 575	N = 557	N = 1654	N = 9817
Age^a^	35(30–41)	37(31–43)	37(30–44)	36(30–42)	34(28–41)	34(28–42)	36(30–43)
*% missing* b	4.5	1.5	19.5	0	0	0	9.7
Female n(%)	501(30)	106(11)	2477(55)	274(48)	65(12)	485(29)	3908(40)
Sexual transmission n(%)^c^	1224(85)	912(99)	-	550(99)	538(98)	1302(99)	8916(97)
*% missing*	11.8	1.5	100	4	1.9	20.8	51.4
Married n(%)	-	-	743 (17)	43(54)	88(16)	544(36)	1418(22)
*% missing*	100	100	0.7	86.3	2.7	9.8	33.4
Education years^d^	10.44(4.9)	13.06(3.5)	6.10(4.4)	7.56(3.9)	11.73(4.1)	11.74(2.6)	8.55(4.9)
% *missing*	47.7	1.5	19.5	0	0	0	14.4
Employed n(%)	786(74)	561(65)	-	338(79)	348(63)	1179(78)	3212(73)
*% missing*	34.9	7.6	100	25.4	0.3	8.6	54.9
CD4 count^a^	181(56–309)	140 (51–210)	133 (55–207)	105 (53–185)	147 (54–254)	82 (32–176)	129 (49–219)
*% missing*	19.7	0	8.6	33.4	14.0	19.4	13.3
LHI n(%)	734(55.7)	742(79.7)	3086(75.8)	347(90.6)	379(79.1)	1143(85.7)	6431(75.5)
% *missing*	19.7	0	8,6	33.4	14.0	19.4	13.3
CD4<200 & no ADI n(%)	607(46.0)	393(42.2)	2211(54.3)	88(22.9)	121(25.3)	412(30.9)	3832(45)
ADI & CD4>200 n(%)	21(1.6)	90(9.7)	101(2.5)	43(11.2)	79(16.5)	80(6.0)	414(4.9)
CD4<200 & ADI n(%)	106(8.0)	259(27.8)	774(19.0)	216(56.4)	179(37.4)	651(48.8)	2185(25.7)

**NOTE**. HAART, highly active antiretroviral therapy; FH-Argentina, Fundación Huésped in Buenos Aires; FA-Chile, Fundación Arriarán in Santiago, GHESKIO-Haiti, Le Groupe Haïtien d'Etude du Sarcome de Kaposi et des Infections Opportunistes in Port-au-Prince; IHSS/HE-Honduras Instituto Hondureño de Seguridad Social and Hospital de Especialidades in Tegucigalpa; INNSZ-Mexico, Instituto Nacional de Ciencias Médicas y Nutrición Salvador Zubirán in Mexico City; and IMTAvH-Peru, Instituto de Medicina Tropical Alexander von Humboldt in Lima; LHI, late HAART initiators.

a Median (interquartile range) reported for all continuous variables except education years.

b Missing values correspond to variables not routinely collected by some sites (e.g. marital status) or values not available within the required time frame (e.g. baseline CD4).

c Information about MSM transmission was only available in Argentina and Mexico. Argentina had 437 MSM (35.9%) and Mexico 312 MSM (65.1%).

d Mean (standard deviation) reported for education years.

### Rates of LHI

**Figure 1:**
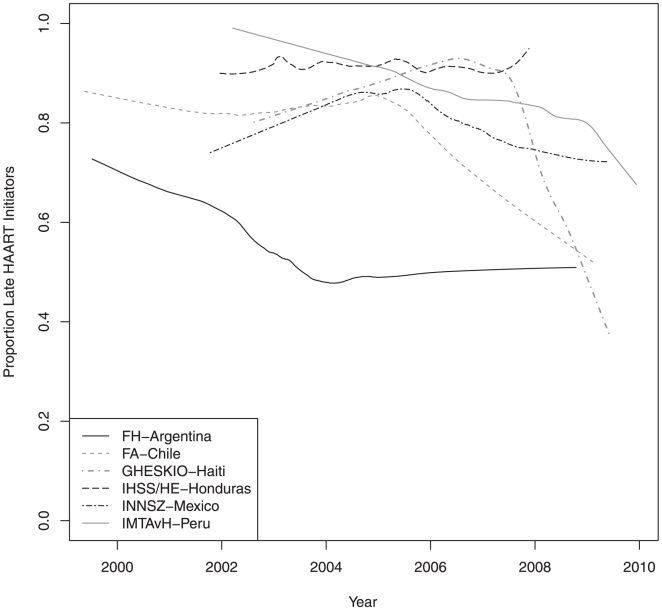
Temporal trends in the proportion of patients classified as late HAART initiators. **NOTE**. FH-Argentina, Fundación Huésped in Buenos Aires, Argentina; FA-Chile, Fundación Arriarán in Santiago, Chile; GHESKIO-Haiti, Le Groupe Haïtien d'Etude du Sarcome de Kaposi et des Infections Opportunistes in Port-au-Prince, Haiti; IHSS/HE-Honduras Instituto Hondureño de Seguridad Social and Hospital de Especialidades in Tegucigalpa, Honduras; INNSZ-Mexico, El Instituto Nacional de Ciencias Médicas y Nutrición Salvador Zubirán in Mexico City, Mexico; and IMTAvH-Peru, El Instituto de Medicina Tropical Alexander von Humboldt in Lima, Perú.

Of 8515 patients with a recorded baseline CD4 cell count, 6431 (76% [95%CI: 74–77]) were LHI: 56% [95%CI: 52–59] in FH-Argentina, 80% [95%CI: 77–82] in FA-Chile, 76% [95%CI: 74–77] in GHESKIO-Haiti, 91%[95%CI: 87–94] in IHSS/HE-Honduras, 79% [95%CI: 75–83]) in INNSZ-Mexico and 86% [95%CI: 84–88] in IMTAvH-Peru. At HAART initiation, 70.4% of patients had CD4<200 (with or without ADI), 30.5% had an ADI (regardless of CD4+ count), and 25.6% had both characteristics. The proportion of LHI over time for each site is shown in Figure 1. For all of the sites except IHSS/HE-Honduras, the proportion of LHI statistically changed over time (*p*<0.001 for FH-Argentina, FA-Chile, GHESKIO-Haiti, and IMTAvH-Peru; *p* = 0.02 for INNSZ-Mexico; and *p* = 0.86 for IHSS/HE-Honduras). Rates of LHI tended to be lower in more recent years for most of the sites. The rates of LHI in INNSZ-Mexico and GHESKIO-Haiti increased in earlier years followed by a decrease in later years. In contrast, in FH-Argentina the rate of LHI decreased in earlier years and stayed roughly constant from 2004 to 2009. Note that 95.2% of patients started HAART with CD4<350 or an ADI.

### Risk factors for LHI

In multivariate logistic analyses including age, sex, years of education, and date of HAART initiation, male sex was associated with an increased risk of LHI in FA-Chile, GHESKIO-Haiti, IMTAvH-Peru, and in the combined site analyses (OR = 2.93, 95% CI 1.59–5.40; OR = 1.27, CI 1.06–1.51; OR = 2.28, CI 1.64–3.18; OR = 1.69, CI 1.24–2.30; respectively; Table 2). Older patients were more likely to be LHI in FH-Argentina and IMTAvH-Peru (OR 1.21 per 10 year increase in age, 95% CI 1.02–1.45; OR 1.20, CI 1.02–1.43; respectively), although this trend was not seen across all cohorts (OR 1.07, 95% CI 0.94–1.21). Higher education was statistically associated with a decreased risk of LHI in FA-Chile (OR 0.92 per additional year of education, CI 0.87–0.98), and similar trends, although not statistically significant, were seen in INNSZ-Mexico, IMTAvH-Peru, and in the combined site analysis. The estimated probability of LHI over time after adjusting for age, sex, and education was similar to the unadjusted trends seen above, except perhaps in FH-Argentina where there appeared to be a stronger recent trend to lower rates of LHI (see Figure S1 of Supporting Information section).

**Table 2:** Multivariate analyses of factors associated with late HAART initiation^a^.

	FH-Argentina	FA-Chile	GHESKIO-Haiti	IHSS/HE-Honduras	INNSZ-México	IMTAvH-Perú	Combined
Age	1.21	0.93	0.95	0.86	1.22	1.20	1.07
(per 10 yrs)	(1.02, 1.45)	(0.76, 1.14)	(0.87, 1.04)	(0.56, 1.32)	(0.94, 1.59)	(1.02, 1.43)	(0.94, 1.21)
*p-value*	0.03	0.49	0.30	0.49	0.13	0.03	0.31
Male	1.19	2.93	1.27	1.59	1.91	2.28	1.69
	(0.83, 1.69)	(1.59, 5.40)	(1.06, 1.51)	(0.68, 3.72)	(0.93, 3.92)	(1.64, 3.18)	(1.24, 2.30)
*p-value*	0.34	<0.001	0.01	0.28	0.08	<0.001	0.001
Education	0.99	0.92	0.99	0.98	0.95	0.94	0.97
(per 1 yr)	(0.95, 1.02)	(0.87, 0.98)	(0.97, 1.01)	(0.88, 1.08)	(0.90, 1.02)	(0.88, 1.01)	(0.95, 1.00)
*p-value*	0.46	0.008	0.48	0.66	0.14	0.07	0.02

**NOTE**. HAART, highly active antiretroviral therapy; FH-Argentina, Fundación Huésped in Buenos Aires, Argentina; FA-Chile, Fundación Arriarán in Santiago, Chile; GHESKIO-Haiti, Le Groupe Haïtien d'Etude du Sarcome de Kaposi et des Infections Opportunistes in Port-au-Prince, Haiti; IHSS/HE-Honduras Instituto Hondureño de Seguridad Social and Hospital de Especialidades in Tegucigalpa, Honduras; INNSZ-México, El Instituto Nacional de Ciencias Médicas y Nutrición Salvador Zubirán in Mexico City, Mexico; and IMTAvH-Perú, El Instituto de Medicina Tropical Alexander von Humboldt in Lima, Perú.

a Data shown are odds ratios (95% confidence intervals). Estimates are adjusted for all variables in the table as well as date of HAART initiation using a logistic regression model.

In secondary multivariate analyses that included other predictors which were recorded at specific sites, employment was associated with lower rates of LHI in FH-Argentina (OR 0.50, 95% CI 0.27–0.92), but not in FA-Chile, IHSS/HE-Honduras, INNSZ-Mexico, or IMTAvH-Peru (p>0.1 for all) (see Table S2 of Supporting Information section). In FH-Argentina, men who have sex with men (MSM) and those whose probable route of infection was heterosexual tended to have lower rates of LHI than those whose probable route of infection was non-sexual (OR = 0.49, 95% CI 0.24–1.00; OR = 0.66, 95% CI 0.32–1.35; respectively); in INNSZ-Mexico, MSM also tended to have lower rates of LHI than heterosexuals, although this was not statistically significant (OR = 0.81, 95% CI 0.41–1.58). Being married had little association with LHI in any of the sites that recorded marital status (OR 0.92, 95% CI 0.73–1.16, p = 0.48; OR 0.58, 95% CI 0.29–1.17, p = 0.13; OR 0.76, 95% CI 0.53–1.07, p = 0.11 for GHESKIO-Haiti, INNSZ-Mexico, and IMTAvH-Peru, respectively).

### Rates of LT and LP

**Figure 2:**
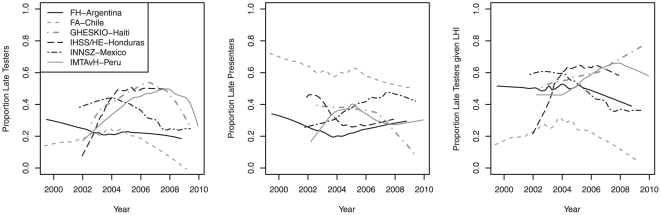
Temporal trends in the proportion of patients classified as late testers and late presenters. **NOTE**. The left and center plots are the corresponding proportions based on the entire cohort population (denominator = all HAART initiators). The right plot shows trends in the proportion of late HAART initiators who were late testers (denominator = all late HAART initiators). Rates of LT (left plot) statistically changed over time for most sites (p<0.001 for all sites except FH-Argentina where p = 0.42). Rates of LP (center plot) also changed over time for most sites (p = 0.11, 0.007, <0.001, 0.01, <0.001, and 0.01 for FH-Argentina, FA-Chile, GHESKIO-Haiti, IHSS/HE-Honduras, INNSZ-Mexico, and IMTAvH-Peru, respectively). FH-Argentina, Fundación Huésped in Buenos Aires, Argentina; FA-Chile, Fundación Arriarán in Santiago, Chile; GHESKIO-Haiti, Le Groupe Haïtien d'Etude du Sarcome de Kaposi et des Infections Opportunistes in Port-au-Prince, Haiti; IHSS/HE-Honduras Instituto Hondureño de Seguridad Social and Hospital de Especialidades in Tegucigalpa, Honduras; INNSZ-Mexico, El Instituto Nacional de Ciencias Médicas y Nutrición Salvador Zubirán in Mexico City, Mexico; and IMTAvH-Peru, El Instituto de Medicina Tropical Alexander von Humboldt in Lima, Perú. LHI, late HAART initators.

Of 6047 LHI with a recorded date of HIV diagnosis, 3331 (55%) were late testers and 2716 (45%) were late presenters, corresponding to 42% and 34% of the 7901 patients in the overall cohort with complete data. The proportion of late HAART initiators who were LT was 50% for FH-Argentina, 23% for FA-Chile, 63% for GHESKIO-Haiti, 56% for IHSS/HE-Honduras, 48% for INNSZ-Mexico, and 59% for IMTAvH-Peru. Temporal trends were seen across sites (Figure 2). Notably, the proportions of late testers and late presenters both dropped substantially in GHESKIO-Haiti in recent years, although in 2007–2008 over 60% of GHESKIO-Haiti's LHI were late testers.

### Risk Factors for LT and LP

Male sex was associated with late presentation and especially late testing for most sites and when all sites were considered together (Table 3; combined OR = 1.51, 95% CI 1.12–2.03, p-value 0.001 for LP; combined OR = 1.76, 95% CI 1.36–2.29, p-value<0.001 for LT). Older age was a risk factor for late testing (OR = 1.17 per 10 year increase in age, 95% CI 1.03–1.34, p-value 0.02), whereas higher education tended to be associated with a decreased risk of late presentation (OR 0.97, 95% CI 0.95–1.00, p-value 0.005).

**Table 3:** Multivariate analyses of risk factors associated with late testers and late presenters^a^.

	FH-Argentina	FA-Chile	GHESKIO-Haiti	IHSS/HE-Honduras	INNSZ-México	IMTAvH-Perú	Combined
**LT**							
Age (10 yr)	1.50	1.08	1.04	0.87	1.28	1.23	1.17
	(1.20–1.88)	(0.86–1.37)	(0.94–1.14)	(0.54–1.41)	(0.95–1.72)	(1.03–1.46)	(1.03–1.34)
*p-value*	0.00	0.47	0.43	0.59	0.10	0.02	0.02
Male	1.44	1.95	1.36	2.23	2.57	2.32	1.76
	(0.90–2.31)	(1.03–3.67)	(1.12–1.64)	(0.89–5.6)	(1.08–6.14)	(1.63–3.30)	(1.36–2.29)
*p-value*	0.12	0.04	0.001	0.08	0.03	0.00	0.00
Education (yr)	1.00	1.00	0.99	0.99	0.95	0.96	1.00
	(0.96–1.05)	(0.99–1.01)	(0.97–1.02)	(0.89–1.11)	(0.88–1.01)	(0.90–1.03)	(1.00–1.01)
*p-value*	0.87	0.27	0.93	0.97	0.13	0.29	0.40
**LP**							
Age (10 yr)	0.99	0.89	0.77	0.87	1.25	1.06	0.95
	(0.78–1.26)	(0.74–1.07)	(0.69–0.86)	(0.54–1.42)	(0.94–1.67)	(0.88–1.27)	(0.81–1.11)
*p-value*	0.96	0.24	0.00	0.58	0.12	0.52	0.53
Male	1.18	2.19	1.12	1.30	1.53	2.12	1.51
	(0.76–1.84)	(1.33–3.63)	(0.90–1.38)	(0.52–3.26)	(0.69–3.41)	(1.46–3.07)	(1.12–2.03)
*p-value*	0.46	0.002	0.30	0.57	0.29	0.00	0.001
Education (yr)	0.96	1.00	0.97	0.96	0.95	0.92	0.97
	(0.92–1.01)	(0.99–1.01)	(0.95–1.00)	(0.85–1.07)	(0.89–1.02)	(0.86–0.98)	(0.95–1.00)
*p-value*	0.10	0.07	0.06	0.44	0.16	0.02	0.005

**NOTE**. FH-Argentina, Fundación Huésped in Buenos Aires, Argentina; FA-Chile, Fundación Arriarán in Santiago, Chile; GHESKIO-Haiti, Le Groupe Haïtien d'Etude du Sarcome de Kaposi et des Infections Opportunistes in Port-au-Prince, Haiti; IHSS/HE-Honduras Instituto Hondureño de Seguridad Social and Hospital de Especialidades in Tegucigalpa, Honduras; INNSZ-Mexico, El Instituto Nacional de Ciencias Médicas y Nutrición Salvador Zubirán in Mexico City, Mexico; and IMTAvH-Peru, El Instituto de Medicina Tropical Alexander von Humboldt in Lima, Perú.

a Data shown are odds ratios (95% confidence intervals). Estimates are adjusted for all variables in the table as well as date of HAART initiation.

Analyses were repeated using multiple imputation techniques to include patients with incomplete data. In general, results were very similar to those reported above (Supporting Information section). The estimated rates of LHI were 56% in FH-Argentina, 80% in FA-Chile, 76% in GHESKIO-Haiti, 89% in IHSS/HE-Honduras, 78% in INNSZ-Mexico, and 86% in IMTAvH-Peru. The only notable changes in multivariate analyses were 1) education was associated with LHI in FH-Argentina (OR = 0.97 per year, 95% CI 0.95–0.99) and 2) the association between age and LHI in IMTAvH-Peru slightly weakened (OR = 1.16 per 10 years, 95% CI 0.98–1.37).

## Discussion

The present study conducted among cohorts in different sites from 6 Latin-American countries shows that a significant proportion of patients starting HAART in this region do so after they have experienced an AIDS-defining illness or when the CD4 cell count is lower than 200, far below the cutoff of 350 cells/mm^3^ that is the recommended by most of the current international guidelines [30]–[32]. The prevalence of late HAART initiation as defined in this study ranges from 56% in FH-Argentina to 91% in IHSS/HE-Honduras with a combined mean of 75%. This finding presents a harsh reality to the current debate on when to start antiretroviral treatment [33], [34]. While recent World Health Organization guidelines [30] have raised the target CD4 cell count at which treatment should be initiated, we found that most patients in our region still initiate treatment at a level which is far from optimal. In contrast to previous studies [20], our study analyzed not only the frequency with which patients present late to care, but as importantly, the frequency at which patients initiate therapy late. It has been assumed from previous studies that the main reason for late HAART initiation is that patients are not diagnosed early enough, often reflecting a failure of the local health systems to implement widespread testing strategies [2], [4], [35]. In our study, however, a surprising finding was that a significant proportion of LHI (nearly 50% in the combined cohort; the proportion of patients who were LHI and were diagnosed more than 6 months before HAART initiation ranged from as high as 80% in FA-Chile, to approximately 40% in GHESKIO-Haiti and IMTAvH-Peru) were in fact diagnosed more than 6 months before starting HAART, and therefore, initiating late was not necessarily due to patients testing for HIV at late stages of disease but to other unexplained reasons. These reasons could include a poor linkage between the testing programs and the access to care facilities [20], [36], [37], need to be further studied. The high rates of late presentation are particularly noteworthy because access to HAART should not be a problem in any of the participating sites, as compared to other reports in Latin America [38], given that each is part of local programs for universal coverage of HAART paid by their respective governments.

Recently, in a large cohort of 14 sites in the USA and Canada (NA-ACCORD), late presentation to care seem to be have been decreasing between 1997 and 2007, based on the change in the median of CD4+ count at presentation to care [39]. In our region, from 2004 to 2009 there appears to be a trend towards fewer patients initiating HAART in advanced disease stages in more recent years. This was particularly apparent in Haiti, where rates of LHI have substantially dropped since 2007. This drop is most likely attributable to a clinic-wide effort to initiate HAART in patients at earlier disease stages and also to the CIPRA study conducted at GHESKIO, which included patients starting HAART with CD4 cell counts that were higher than 200 cells/mm^3^ [15]. Furthermore, GHESKIO has an integrated testing and treatment center, which facilitates linkages in care [15], [40], [41]. The high rates of LHI in the earlier study years could be attributed in part to local guidelines containing late thresholds for initiating HAART [41]–[45]. Trends towards lower rates of LHI in recent years probably reflect in part a change in guidelines. Nevertheless, despite these trends, the prevalence of LHI in the most recent years still remains unacceptably high.

The outstanding risk factor for LHI in the multivariate analysis was male sex. Older age and less education were also associated with higher rates of LHI in many of the sites. Even though the AIDS epidemic in most of the participating countries is driven by MSMs, in the sites where these data were available (INNSZ-Mexico and FH-Argentina) MSMs tended to have a somewhat lower risk of being LHI. In the combined analysis of all cohorts, the same factors (male sex and older age) were significantly associated with late testing (recent diagnosis in LHI) and with late presentation (diagnosis more than 6 months before HAART initiation). These risk factors are similar to those reported in other studies for patients diagnosed in advanced stages [8].

One limitation of our study is the number of missing CD4 cell counts at HAART initiation from some of the sites. The analysis presented excluded those patients without data, which may bias the results. However, we obtained similar results using multiple imputation techniques which allow the inclusion of all patients initiating HAART. Another potential limitation is that we may have had insufficient power to detect some risk factors for LHI due to there being very few NLHI in some sites. The heterogeneity between rates of LHI at the different sites is also noteworthy, and suggests that there are important differences between clinic settings, perhaps in traditions, culture, HIV stigma, testing services, education, or something else. Unfortunately, with only six sites, we do not have sufficient data to investigate site-specific factors associated with LHI.

It is important to emphasize that our study did not measure the rate of late testing, but rather the proportion of patients who started HAART who were considered late testers. Of course, these patients represent only those who made it to care and received treatment. Our study does not consider persons in late stages of disease who do not know their HIV status, or diagnosed patients in late stages of disease who have not started treatment.

In conclusion our study shows a high rate of HAART initiation at CD4 cell counts <200 cell/mm^3^ or with an AIDS-defining illness in Latin-American and the Caribbean, which is substantially below the current cutoff recommended in international guidelines [30]–[32]. This observation is mostly explained by late HIV diagnosis however, a significant proportion of patients across all sites that meet criteria for treatment initiation experience considerable delays between the time of HIV diagnosis and the initiation of HAART. Avoiding this delay by improving the linkage between testing and treatment, as well as promoting early diagnosis of HIV, should result in better immunologic status at HAART initiation and better outcomes.

## Supporting Information

Figure S1 **Probability of being a LHI over time adjusting for age, sex, and years of education.** **NOTE**. 95% confidence intervals are reported. The adjusted rates of LHI statistically changed over time for most sites (p<0.001 for all sites except INNSZ-Mexico, p = 0.05, and IHSS/HE-Honduras, p = 0.95). FH-Argentina, Fundación Huésped in Buenos Aires, Argentina; FA-Chile, Fundación Arriarán in Santiago, Chile; GHESKIO-Haiti, Le Groupe Haïtien d'Etude du Sarcome de Kaposi et des Infections Opportunistes in Port-au-Prince, Haiti; IHSS/HE-Honduras Instituto Hondureño de Seguridad Social and Hospital de Especialidades in Tegucigalpa, Honduras; INNSZ-Mexico, El Instituto Nacional de Ciencias Médicas y Nutrición Salvador Zubirán in Mexico City, Mexico; and IMTAvH-Peru, El Instituto de Medicina Tropical Alexander von Humboldt in Lima, Perú. LHI, late HAART initators.
(EPS)

Table S1 **Number of patients by Calendar year of time of HAART initiation by site.** **NOTE**. HAART, highly active antiretroviral therapy; FH-Argentina, Fundación Huésped in Buenos Aires; FA-Chile, Fundación Arriarán in Santiago, GHESKIO-Haiti, Le Groupe Haïtien d'Etude du Sarcome de Kaposi et des Infections Opportunistes in Port-au-Prince; IHSS/HE-Honduras Instituto Hondureño de Seguridad Social and Hospital de Especialidades in Tegucigalpa; INNSZ-Mexico, Instituto Nacional de Ciencias Médicas y Nutrición Salvador Zubirán in Mexico City; and IMTAvH-Peru, Instituto de Medicina Tropical Alexander von Humboldt in Lima; LHI, late HAART initiators.
(DOCX)

Table S2 **Odds ratios (95% confidence intervals) for late HAART initiation using multiple imputation^a^.** **NOTE**. FH-Argentina, Fundación Huésped in Buenos Aires, Argentina; FA-Chile, Fundación Arriarán in Santiago, Chile; GHESKIO-Haiti, Le Groupe Haïtien d'Etude du Sarcome de Kaposi et des Infections Opportunistes in Port-au-Prince, Haiti; IHSS/HE-Honduras Instituto Hondureño de Seguridad Social and Hospital de Especialidades in Tegucigalpa, Honduras; INNSZ-Mexico, El Instituto Nacional de Ciencias Médicas y Nutrición Salvador Zubirán in Mexico City, Mexico; and IMTAvH-Peru, El Instituto de Medicina Tropical Alexander von Humboldt in Lima, Perú. ^a^Data shown are odds ratios (95% confidence intervals). Estimates are adjusted for all variables in the table as well as date of HAART initiation.
(DOCX)
