# Supplementary material for: Cross-Sectional Analysis of Late HAART Initiation in Latin America and the Caribbean: Late Testers and Late Presenters
Source: PLoS One. 2011 May 26;6(5):e20272. doi: 10.1371/journal.pone.0020272 (PMC3102699; doi:10.1371/journal.pone.0020272)
Supplement: Table S1 — Number of patients by Calendar year of time of HAART initiation by site. NOTE. HAART, highly active antiretroviral therapy; FH-Argentina, Fundación Huésped in Buenos Aires; FA-Chile, Fundación Arriarán in Santiago, GHESKIO-Haiti, Le Groupe Haïtien d'Etude du Sarcome de Kaposi et des Infections Opportunistes in Port-au-Prince; IHSS/HE-Honduras Instituto Hondureño de Seguridad Social and Hospital de Especialidades in Tegucigalpa; INNSZ-Mexico, Instituto Nacional de Ciencias Médicas y Nutrición Salvador Zubirán in Mexico City; and IMTAvH-Peru, Instituto de Medicina Tropical Alexander von Humboldt in Lima; LHI, late HAART initiators. (DOCX) [file pone.0020272.s002.docx]

**Supporting Information**

**Table S1.** Number of patients by Calendar year of time of HAART initiation by site

|  | **FH-Argentina** | **FA-Chile** | **GHESKIO-Haiti** | **IHSS/HE-Honduras** | **INNSZ-México** | **IMTAvH-Perú** | **Combined** |
| --- | --- | --- | --- | --- | --- | --- | --- |
|  | **N=1642** | **N=931** | **N=4458** | **N=575** | **N=557** | **N=1654** | **N=9817** |
| Calendar year n(%) |  |  |  |  |  |  |  |
| 1999 | 4(0.2) | 24(2.6) | - | - | - | - | 28(0.3) |
| 2000 | 159(9.7) | 65(6.9) | - | 1(0.1) | - | 9(0.5) | 234(2.4) |
| 2001 | 204(12.4) | 82(8.8) | - | 5(0.9) | 2(0.3) | 15(0.9) | 308(3.1) |
| 2002 | 182(11.1) | 153(16.4) | - | 36(6.2) | 39(7) | 20(1.2) | 430(4.3) |
| 2003 | 385(23.4) | 106(11.3) | 552(12.3) | 132(22.9) | 71(12.7) | 44(2.7) | 1290(13.1) |
| 2004 | 226(13.8) | 146(15.6) | 559(12.5) | 96(16.7) | 68(12.2) | 212(12.8) | 1307(13.3) |
| 2005 | 204(12.4) | 116(12.4) | 218(4.9) | 92(16) | 60(10.7) | 301(18.2) | 991(10.1) |
| 2006 | 117(7.1) | 90(9.7) | 338(7.6) | 85(14.8) | 62(11.1) | 217(13.1) | 909(9.3) |
| 2007 | 61(3.7) | 70(7.5) | 719(16.1) | 85(14.8) | 80(14.3) | 247(14.9) | 1262(12.9) |
| 2008 | 75(4.6) | 33(3.5) | 875(19.6) | 43(7.5) | 77(13.8) | 266(16.1) | 1369(13.9) |
| 2009 | 25(1.5) | 44(4.7) | 1197(26.8) | - | 91(16.3) | 249(15.0) | 1606(16.4) |
| 2010 | - | 2(0.2) | - | - | 7(1.2) | 74(4.5) | 83(0.9) |

**Legend Table S1**. HAART, highly active antiretroviral therapy; FH-Argentina, Fundación Huésped in Buenos Aires; FA-Chile, Fundación Arriarán in Santiago, GHESKIO-Haiti, Le Groupe Haïtien d'Etude du Sarcome de Kaposi et des Infections Opportunistes in Port-au-Prince; IHSS/HE-Honduras Instituto Hondureño de Seguridad Social and Hospital de Especialidades in Tegucigalpa; INNSZ-Mexico, Instituto Nacional de Ciencias Médicas y Nutrición Salvador Zubirán in Mexico City; and IMTAvH-Peru, Instituto de Medicina Tropical Alexander von Humboldt in Lima; LHI, late HAART initiators.
