# Supplementary material for: Cross-Sectional Analysis of Late HAART Initiation in Latin America and the Caribbean: Late Testers and Late Presenters
Source: PLoS One. 2011 May 26;6(5):e20272. doi: 10.1371/journal.pone.0020272 (PMC3102699; doi:10.1371/journal.pone.0020272)
Supplement: Table S2 — Odds ratios (95% confidence intervals) for late HAART initiation using multiple imputationa. NOTE. FH-Argentina, Fundación Huésped in Buenos Aires, Argentina; FA-Chile, Fundación Arriarán in Santiago, Chile; GHESKIO-Haiti, Le Groupe Haïtien d'Etude du Sarcome de Kaposi et des Infections Opportunistes in Port-au-Prince, Haiti; IHSS/HE-Honduras Instituto Hondureño de Seguridad Social and Hospital de Especialidades in Tegucigalpa, Honduras; INNSZ-Mexico, El Instituto Nacional de Ciencias Médicas y Nutrición Salvador Zubirán in Mexico City, Mexico; and IMTAvH-Peru, El Instituto de Medicina Tropical Alexander von Humboldt in Lima, Perú. aData shown are odds ratios (95% confidence intervals). Estimates are adjusted for all variables in the table as well as date of HAART initiation. (DOCX) [file pone.0020272.s003.docx]

**Supporting Information**

**Table S2.** Odds ratios (95% confidence intervals) for late HAART initiation using multiple imputation^a^

|  | **FH-Argentina** | **FA-Chile** | **GHESKIO-Haiti** | **IHSS/HE-Honduras** | **INNSZ-Mexico** | **IMTAvH-Peru** | **Combined** |
| --- | --- | --- | --- | --- | --- | --- | --- |
| Age (per 10yrs) | 1.19 | 0.95 | 0.97 | 0.98 | 1.24 | 1.16 | 1.07 |
|  | (1.04-1.35) | (0.79-1.14) | (0.89-1.06) | (0.69-1.40) | (0.98-1.37) | (0.98-1,37) | (0.97-1.19) |
| p-value | 0.004 | 0.58 | 0.53 | 0.93 | 0.07 | 0.09 | 0.16 |
| Male | 1.15 | 2.57 | 1.22 | 1.35 | 1.69 | 2.01 | 1.52 |
|  | (0.90-1.47) | (1.53-4.34) | (1.04-1.43) | (0.72-2.52) | (0.85-3.35) | (1.46-2.76) | (1.18-1.95) |
| p-value | 0.51 | <0.001 | 0.02 | 0.35 | 0.14 | <0.001 | 0.002 |
| Education | 0.97 | 0.92 | 1.01 | 0.99 | 0.95 | 0.95 | 0.97 |
| (per 1yr) | (0.95-0.99) | (0.86-0.98) | (0.99-1.02) | (0.90-1.08) | (0.89-1.01) | (0.90-1.02) | (0.95-1.00) |
| p-value | 0.18 | 0.005 | 0.59 | 0.84 | 0.10 | 0.16 | 0.05 |

**Legend Table S2**. FH-Argentina, Fundación Huésped in Buenos Aires, Argentina; FA-Chile, Fundación Arriarán in Santiago, Chile; GHESKIO-Haiti, Le Groupe Haïtien d'Etude du Sarcome de Kaposi et des Infections Opportunistes in Port-au-Prince, Haiti; IHSS/HE-Honduras Instituto Hondureño de Seguridad Social and Hospital de Especialidades in Tegucigalpa, Honduras; INNSZ-Mexico, El Instituto Nacional de Ciencias Médicas y Nutrición Salvador Zubirán in Mexico City, Mexico; and IMTAvH-Peru, El Instituto de Medicina Tropical Alexander von Humboldt in Lima, Perú.

^a^Data shown are odds ratios (95% confidence intervals). Estimates are adjusted for all variables in the table as well as date of HAART initiation
